# Stereolithography-printed Bi_2_O_3_-reinforced photopolymer nanocomposite samples with antibacterial and radiation shielding properties

**DOI:** 10.1007/s00411-026-01235-6

**Published:** 2026-06-24

**Authors:** İlkan Özkan, Nadiye Merve Aydın, Ferdi Akman

**Affiliations:** 1 Department of Materials Science and Engineering, Faculty of Engineering, Adana Alparslan Türkeş Science and Technology University, Adana, Türkiye Turkey; 2 Department of Materials Science, Institute of Graduate School, Adana Alparslan Türkeş Science and Technology University, Adana, Türkiye Turkey; 3 Department of Property Protection and Security, Program of Occupational Health and Safety, Vocational School of Social Sciences, Bingöl University, Bingöl, Türkiye Turkey

**Keywords:** Bi_2_O_3_, Stereolithography, Antibacterial activity, Electrical properties, Radiation shielding

## Abstract

Stereolithography (SLA) is an additive manufacturing technique that allows the production of polymer composite samples with high accuracy and smooth surface quality. This makes SLA suitable for functional surface applications. In this study, photopolymer nanocomposite surfaces reinforced with bismuth oxide (Bi_2_O_3_) nanoparticles were produced by SLA and evaluated in terms of surface structure, antibacterial activity, electrical properties, electromagnetic shielding, and ionizing radiation shielding performance. Bi_2_O_3_ nanoparticles were added to a commercial UV-curable photopolymer resin at contents of 1, 3, and 5 wt% and printed layer by layer with a layer thickness of 50 μm. Surface structure and particle distribution were examined by SEM–EDS. The results showed good particle distribution at low Bi_2_O_3_ contents, while particle accumulation occurred at higher contents due to settling during printing. Antibacterial activity was tested according to ISO 22,196, and all Bi_2_O_3_-reinforced surfaces showed more than 99% reduction against Escherichia coli and Staphylococcus aureus after 24 h. Surface resistivity measurements performed according to TS EN 1149-1 showed values higher than 10^12^ Ω, indicating strong electrical insulation. Electromagnetic shielding effectiveness values remained below 5 dB in the 8–12 GHz frequency range. In addition, ionizing radiation shielding parameters, including mass attenuation coefficient, linear attenuation coefficient, half-value layer, and radiation protection efficiency, were theoretically calculated using WinXCOM and EpiXS. The results showed that increasing Bi_2_O_3_ content improved photon attenuation, especially at low energies. Overall, Bi2O_3_-reinforced SLA nanocomposites provide strong antibacterial performance, electrical insulation, and improved ionizing radiation shielding, making them suitable for multifunctional surface applications.

## Introduction

Additive manufacturing has gained increasing attention in recent years due to its ability to produce complex geometries with high precision, reduced material waste, and high design flexibility. Among the different additive manufacturing techniques, stereolithography (SLA) has become particularly important because it enables the fabrication of polymer-based components with smooth surface quality and high dimensional accuracy through the photopolymerization of liquid resins (Gardner et al., 2016; Aktitiz et al. 2020; Singh et al., 2020). These advantages have led to the widespread use of SLA in applications where surface quality and geometric accuracy are critical, including biomedical devices, automotive components, and functional surface coatings (Shinde et al., 2020; Chiulan et al. 2022).

With the rapid development of SLA technology, research efforts have increasingly focused on improving the functional properties of photopolymer-based materials. One common approach is the incorporation of nanoparticles into photopolymer resins to enhance mechanical, electrical, thermal, or biological performance. Carbon-based fillers such as carbon nanotubes, graphene oxide, and reduced graphene oxide have been extensively studied due to their ability to improve mechanical strength and electrical behaviour in additively manufactured polymers (Aumnate et al. 2018; Podsiadły et al. 2021; Shah et al., 2023). In addition to carbon-based materials, metal and metal oxide nanoparticles have also been introduced into photopolymer systems to achieve multifunctional performance, including improved thermal stability and surface functionality (Ingrosso et al. 2015; Qiu et al. 2015; V-Niño et al., 2020). However, many of these studies report common challenges such as nanoparticle agglomeration, sedimentation during printing, and reduced print quality at higher filler contents, particularly in low-viscosity SLA resins.

Beyond mechanical and electrical improvements, the development of antibacterial polymer composites has become an important research topic, especially for applications in medical devices and hygienic surfaces. Various inorganic fillers, including silver, zinc oxide, copper oxide, and titanium dioxide, have been shown to provide strong antibacterial activity when incorporated into polymer matrices (Abatay et al., 2015; Olmos et al., 2021). Graphene-based polymer nanocomposites have also been reported to exhibit antibacterial behaviour through physical interactions and oxidative stress mechanisms (Díez-Pascual et al., 2021). Despite their effectiveness, some commonly used antibacterial fillers raise concerns related to ion release, cytotoxicity, or long-term stability, which has motivated the search for alternative materials.

In this context, bismuth oxide (Bi_2_O_3_) has attracted growing interest as functional inorganic filler. Bi_2_O_3_ is characterized by its high atomic number and high density, which contribute to strong interactions with high-energy photons, while also exhibiting effective antibacterial activity with relatively low toxicity compared to conventional antibacterial agents (Geoffrion et al. 2021; Pant et al. 2022). Previous studies have shown that Bi_2_O_3_-containing materials can provide antibacterial functionality as well as radiation-related performance, particularly in systems designed for ionizing radiation attenuation (Liu and Yang 2021; Maestre and Santos 2023). Nevertheless, the integration of Bi_2_O_3_ into photopolymer-based SLA systems has been scarcely reported, and its multifunctional behaviour in such systems remains insufficiently explored. Moreover, although electromagnetic shielding has been widely studied in polymer composites, the performance of Bi_2_O_3_-filled polymer systems remains limited due to the insulating nature of both the filler and the polymer matrix. This highlights the importance of clearly distinguishing between electromagnetic shielding and ionizing radiation shielding when evaluating the functional performance of Bi_2_O_3_-based composites.

Against this background, the present study focused on the fabrication of Bi_2_O_3_-reinforced photopolymer nanocomposite surfaces using SLA and their systematic evaluation in terms of surface morphology, antibacterial activity, surface resistivity, electromagnetic shielding effectiveness, and ionizing radiation shielding parameters. By combining experimental characterization with theoretical radiation shielding calculations, this work aimed to provide a comprehensive understanding of the multifunctional performance, as well as the limitations, of Bi_2_O_3_-filled SLA nanocomposites for advanced surface applications.

## Materials and methods

### Materials

**Fig. 1:**
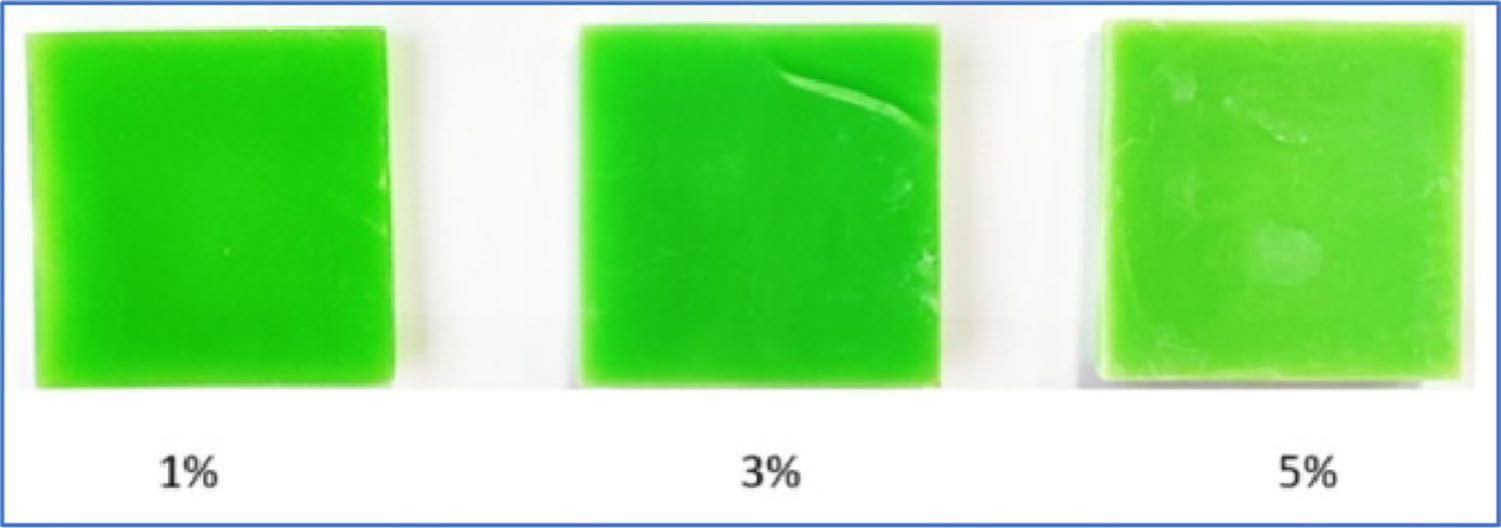
SLA-printed photopolymer composite samples with 1%, 3%, and 5% Bi_2_O_3_ reinforcement

In this study, a commercial standard photopolymer resin supplied by Anycubic (Guangdong, China) was used as the matrix material. The resin is based on a UV-curable system operating at a wavelength of 405 nm and is characterized by low viscosity and good flow behaviour. It was selected due to its full compatibility with SLA-type 3D printers, its capability to produce high-resolution features, and its suitability for forming a uniform and homogeneous structure. The resin has a green coloration and is commonly preferred for the fabrication of precision components. Owing to its photopolymeric nature, effective cross-linking occurs during the layer-by-layer manufacturing process, leading to printed parts with sufficient mechanical integrity. Representative images of the composite samples containing 1, 3, and 5 wt% Bi_2_O_3_ reinforcement is shown in Fig. 1.

The main physical and mechanical properties of the resin used in this study are listed in Table 1.

**Table 1:** Properties of the used resin

Property	Value
Viscosity (25 °C)	552 mPa·s
Tensile Strength	23.4 MPa
UV Curing Wavelength	405 nm
Liquid Density	1.100 g/cm^3^
Solid Density	1.184 g/cm^3^
Elongation at Break	14.2%
Hardness	Shore 79 D
Layer Thickness	50 microns
Layer Curing Time	5–15 s
Bottom Layer Curing Time	20–60 s
Softening Temperature	65–70 °C

The used reinforcing phase in this study consisted of Bi_2_O_3_ nanoparticles with a purity of 99.99%. The nanoparticles are yellow in colour and have an average particle size in the range of 75–195 nm with a nearly spherical morphology. They exhibit a specific surface area of 4.0–6.0 m^2^·g^-1^, a true density of 9.2 g·cm^-3^, a molecular weight of 466 g·mol^-1^, and a melting point of 810 °C. The physical and chemical properties of the Bi_2_O_3_ nanoparticles were provided by the manufacturer (Nanografi Co., Ankara, Türkiye). These characteristics make Bi_2_O_3_ a suitable filler for applications requiring electrical insulation and electromagnetic wave transmission, mainly due to its high atomic number and high density. In addition, Bi_2_O_3_ was selected for this study because of its well-documented antibacterial activity reported in the literature. The spherical particle shape and relatively narrow particle size distribution are advantageous for achieving a uniform dispersion within the photopolymer matrix, which contributes to improved composite performance.

Isopropyl alcohol (IPA, 99% purity) was used as a cleaning agent to remove uncured resin residues from the surface of the printed samples after fabrication. Following the printing process, the samples were washed in an IPA bath and then allowed to dry under ambient conditions to ensure complete removal of residual solvent. Finally, all samples were subjected to post-curing under 405 nm UV light for 15 min to complete the polymerization process and to standardize the specimens prior to testing.

## Methods

### Stereolithography (SLA)

Stereolithography (SLA) is an additive manufacturing technique based on the layer-by-layer photopolymerization of liquid resin using a controlled ultraviolet (UV) light source. In this method, a photosensitive resin is selectively cured through localized exposure to UV radiation, inducing cross-linking reactions that transform the liquid resin into a solid structure with high dimensional accuracy and surface quality. Due to its ability to produce components with fine feature resolution and smooth surface morphology, SLA is particularly suitable for applications requiring precise geometries and homogeneous microstructures.

In the present study, all nanocomposite specimens were fabricated using an SLA-type 3D printer operating at a wavelength of 405 nm. Prior to printing, Bi_2_O_3_ nanoparticles were incorporated into the photopolymer resin at predetermined weight fractions. To ensure uniform nanoparticle dispersion and to minimize agglomeration, the nanoparticle-reinforced resins were mechanically mixed at 2000 revolutions per minute (rpm) for approximately 20 min using a glass-head mechanical stirrer. The homogenized resin mixtures were then transferred into the printer resin tank and immediately used for fabrication to prevent sedimentation.

The test specimens were designed using a computer-aided design (CAD) environment, and their geometries were determined according to the dimensional requirements of subsequent characterization and testing standards. The printing process was conducted under constant environmental conditions to ensure reproducibility. Following completion of the printing process, the fabricated parts were carefully removed from the build plate.

Post-processing steps were applied to eliminate uncured resin residues and to stabilize the printed structures. Initially, the specimens were washed in a 99% IPA bath for 10 min using an automated washing system. This step effectively removed residual uncured resin from the surface and internal features of the printed parts. After washing, the samples were allowed to air-dry to ensure complete evaporation of IPA.

Subsequently, a final UV curing process was performed to enhance the degree of polymer cross-linking and to improve the mechanical and structural stability of the nanocomposites. The specimens were exposed to UV light at a wavelength of 405 nm for 15 min using a dedicated UV curing unit. This post-curing stage is critical in SLA-fabricated parts, as it ensures full polymerization throughout the structure and minimizes potential variations in material properties caused by incomplete curing during the printing stage.

The combined use of controlled nanoparticle dispersion, optimized printing parameters, and standardized post-processing steps enabled the production of homogeneous Bi_2_O_3_-reinforced photopolymer nanocomposites suitable for subsequent morphological, radiation shielding, electromagnetic, and antibacterial performance evaluations.

### Scanning electron microscopy and energy dispersive X-ray spectroscopy (SEM–EDS)

The surface morphology and elemental composition of the fabricated nanocomposite samples were examined using scanning electron microscopy (SEM) combined with energy dispersive X-ray spectroscopy (EDS). All analyses were carried out using a FEI Quanta 650 field emission scanning electron microscope (Thermo Fisher Scientific Co., Massachusetts, USA).

Since both the photopolymer matrix and the Bi_2_O_3_ nanoparticles exhibit insulating behaviour, the sample surfaces were coated with a thin conductive layer prior to SEM observation. A gold–palladium (Au/Pd, 60/40%) coating with a thickness of approximately 5–10 nm was deposited using a Quorum Q150RS sputter coater (Quorum Technologies Co., East Sussex, UK) under an argon atmosphere. This coating minimized surface charging effects and improved image quality during SEM analysis.

SEM imaging was used to investigate the surface morphology of the nanocomposites, including particle size, particle shape, and the dispersion state of Bi_2_O_3_ nanoparticles within the photopolymer matrix. EDS analysis was performed to determine the elemental composition of the samples and to verify the presence and distribution of the constituent elements, including bismuth (Bi), oxygen (O), carbon (C), and other elements originating from the photopolymer resin.

### Electromagnetic shielding effectiveness measurements

According to literature, the electromagnetic shielding performance of Bi_2_O_3_ particles has not been extensively investigated. This is mainly attributed to the inherently low electrical conductivity of Bi_2_O_3_ compared to conventional conductive fillers commonly used in electromagnetic shielding applications. Nevertheless, in order to provide a complete functional characterization of the fabricated nanocomposites, electromagnetic shielding effectiveness (SE) measurements were included in the present study.

**Fig. 2:**
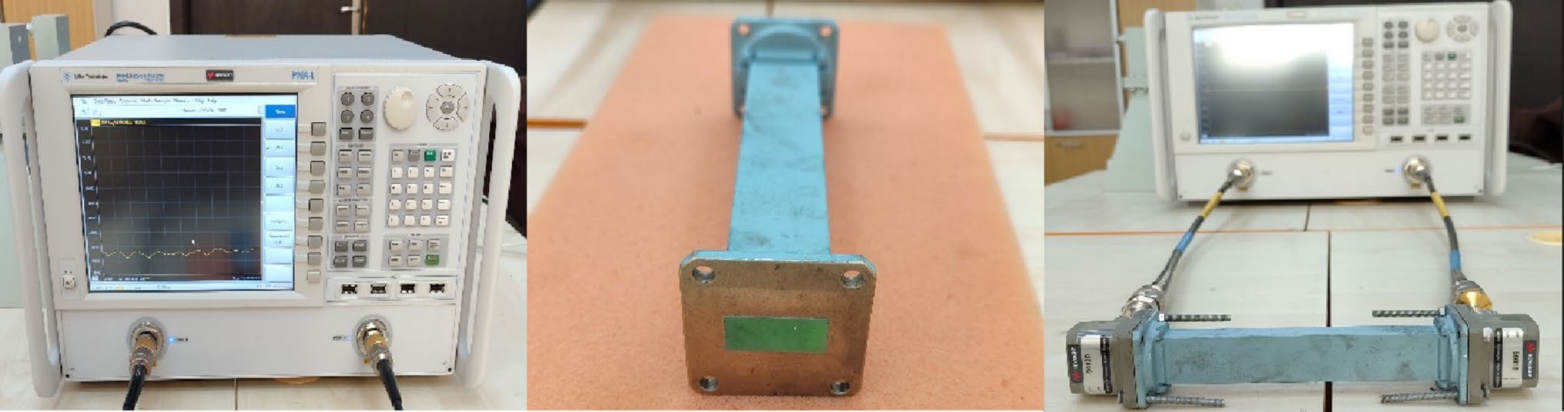
Agilent N5234A PNA-L network analyzer and test setup

Electromagnetic shielding tests were carried out at the Department of Electrical and Electronics Engineering, İskenderun Technical University, İskenderun, Türkiye. The measurements were performed using an Agilent N5234A PNA-L vector network analyzer, a Maury X101A6 waveguide, and a Keysight X281A coaxial-to-waveguide adapter. The measurement system was calibrated prior to testing using a Keysight 85,056 A calibration kit (Keysight Technologies Co., California, USA). A schematic illustration of the experimental setup and the measurement equipment is shown in Fig. 2.

The nanocomposite specimens were fabricated with thicknesses of 4, 7, and 10 mm, and their lateral dimensions were adjusted to fit the waveguide aperture (10.16 mm × 22.86 mm). Transmission parameters (S_12_) were recorded both with and without the samples in the X-band frequency range (8–12 GHz) at 1,001 discrete frequency points. The shielding effectiveness (SE) values, expressed in decibels (dB), were calculated using Eq. (1), which relates the transmitted electromagnetic power in the presence and absence of the specimen (Liang et al. 2017):

1 $$\:\begin{array}{c}SE\left(dB\right)=20{\mathrm{log}}_{10}\left(\frac{{E}_{t}}{{E}_{i}}\right)\:\end{array}$$

where *E*_*t*_ represents the transmitted wave intensity measured with the sample in place, and *E*_*i*_ corresponds to the incident wave intensity measured without the sample.

### Antibacterial activity tests

Evaluation of antibacterial activity is essential for assessing the suitability of the fabricated nanocomposites for potential applications, particularly in medical devices and hospital environments where exposure to bacterial contamination is inevitable. Since radiation shielding materials are often intended for use in such settings, antibacterial performance was included as a key characterization parameter in this study.

Antibacterial tests were carried out in accordance with the ISO 22,196 standard (ISO, 2011). This standard is commonly used to determine antibacterial activity on plastic and other non-porous surfaces. *Staphylococcus aureus* and *Escherichia coli* were selected as representative Gram-positive and Gram-negative bacteria, respectively.

Test specimens with dimensions of 5 × 5 cm were prepared in compliance with the standard and fabricated simultaneously with the other experimental samples to ensure consistency. Prior to testing, all specimens were sterilized by immersion in 70% ethanol. Subsequently, 0.4 mL of bacterial suspension was inoculated onto the surface of each sample, and the inoculated area was covered with a sterile polyethylene film measuring 4 × 4 cm. The samples were then placed in sterile Petri dishes and incubated at 35 °C for 24 h.

After the incubation period, bacterial recovery was performed by transferring the specimens to Petri dishes containing Plate Count Agar (PCA), followed by incubation to enable colony growth and quantification. Control samples without Bi_2_O_3_ reinforcement were subjected to the same procedure under identical conditions.

### Surface resistivity measurements

Surface resistivity measurements were carried out in accordance with the EN 1149-1 standard, which defines a method for determining surface resistance as an indicator of electrostatic discharge behaviour of materials (CEN 2006). All measurements were performed under controlled environmental conditions at a temperature of 25 °C and a relative humidity of 50%.

For each Bi_2_O_3_ reinforcement level (1, 3, and 5 wt%), surface resistivity measurements were repeated five times, and the average values were calculated to ensure measurement reliability. All measurements were carried out using an ELME Multimeg digital megohmmeter (Eurosaft S.r.l., Milan, Italy). Proper electrical contact between the electrodes and the specimen surface was ensured throughout the tests. Surface resistivity values were recorded in ohms per square (Ω/sq).

To improve repeatability and measurement accuracy, the electrode surfaces were cleaned prior to each measurement, and a consistent contact pressure was applied during testing. Based on the measured surface resistivity values, the samples were classified as conductive, dissipative, or insulating based on commonly accepted surface resistivity ranges reported in the literature. The classification ranges used in this study are summarized in Table 2.

**Table 2:** Surface resistivity classification of materials (Groop et al. 2003)

Classification	Surface resistivity (Ω/sq)
Conductive	< 10⁵
Dissipative	10⁵ – 10¹²
Insulating	> 10¹²

### Ionizing radiation shielding performance

The ionizing radiation shielding performance of the fabricated nanocomposites was evaluated theoretically using WinXCOM (Gerward et al. 2001) and EpiXS (Hila et al. 2021) software. The calculations were performed based on the elemental composition and density of each sample. The weight fractions of the constituent elements (H, C, O, P, and Bi) and the corresponding density values were used as input parameters for all calculations. The density values were determined based on the Archimedes principle using the water displacement method. The mass of the samples was measured using a precision balance, and the sample volume was calculated from the increase in water level in a graduated cylinder. The chemical contents and densities of the studied samples summarized in Table 3.

**Table 3:** Chemical content and density of the studied samples; wt - weight

Sample	Chemical content (wt, %)					Density (g cm^− 3^)
	H	C	O	*P*	Bi	
0% Bi_2_O_3_	7.5140	60.6588	31.5681	0.2591	0.0000	0.8500
1% Bi_2_O_3_	7.4389	60.0525	31.3555	0.2565	0.8966	0.8578
3% Bi_2_O_3_	7.2885	58.8383	30.9299	0.2513	2.6920	0.8737
5% Bi_2_O_3_	7.1384	57.6263	30.5049	0.2461	4.4843	0.8903

The mass attenuation coefficient (µ/ρ) of each sample was computed over a photon energy range of 0.015–15 MeV. The mathematical expression of this parameter, determined according to the mixture rule, is presented below (Eq. 2).

2 $$\:\raisebox{1ex}{$\mu\:$}\!\left/\:\!\raisebox{-1ex}{$\rho\:$}\right.={\sum\:}_{i}{W}_{i}{\left(\raisebox{1ex}{$\mu\:$}\!\left/\:\!\raisebox{-1ex}{$\rho\:$}\right.\right)}_{i}\:\:$$

Where *W*_*i*_ represents the weight fraction of the *i*^*th*^ element.

The linear attenuation coefficient (*µ*) was obtained using the sample density (*ρ*) according to Eq. 3.

3 $$\:\begin{array}{c}\mu\:=\left(\frac{{\upmu\:}}{{\uprho\:}}\right)\rho\:\:\end{array}$$

To quantify shielding efficiency in thickness form, the half-value layer (HVL) was calculated from the linear attenuation coefficient derived from the exponential attenuation relationship used in radiation shielding analysis (Eq. 4).

4 $$\:\begin{array}{c}HVL=\frac{\mathrm{ln}\left(2\right)}{\mu\:}\:\:\:\:\:\end{array}$$

The radiation protection efficiency (RPE) was calculated to quantify shielding performance as a function of photon energy and sample thickness using Eq. 5.

5 $$\:\begin{array}{c}RPE\:\left(\mathrm{\%}\right)=\left(1-\frac{I}{{I}_{0}}\right)=\left(1-{e}^{-\mu\:x}\right)\:\:\:\:\:\:\:\end{array}$$

where *I*_*0*_ and *I* represent the incident and transmitted photon intensities, respectively, and *x* is the sample thickness.

RPE calculations were performed for sample thicknesses of 0.4 cm, 0.7 cm, and 1.0 cm, and their variation with photon energy was analyzed. The calculated parameters (*µ/ρ*,* µ*,* HVL*,* RPE*) were used to evaluate the influence of Bi_2_O_3_ reinforcement on the ionizing radiation attenuation behaviour of the nanocomposites.

## Results and discussion

### Morphological structure analysis

**Fig. 3:**
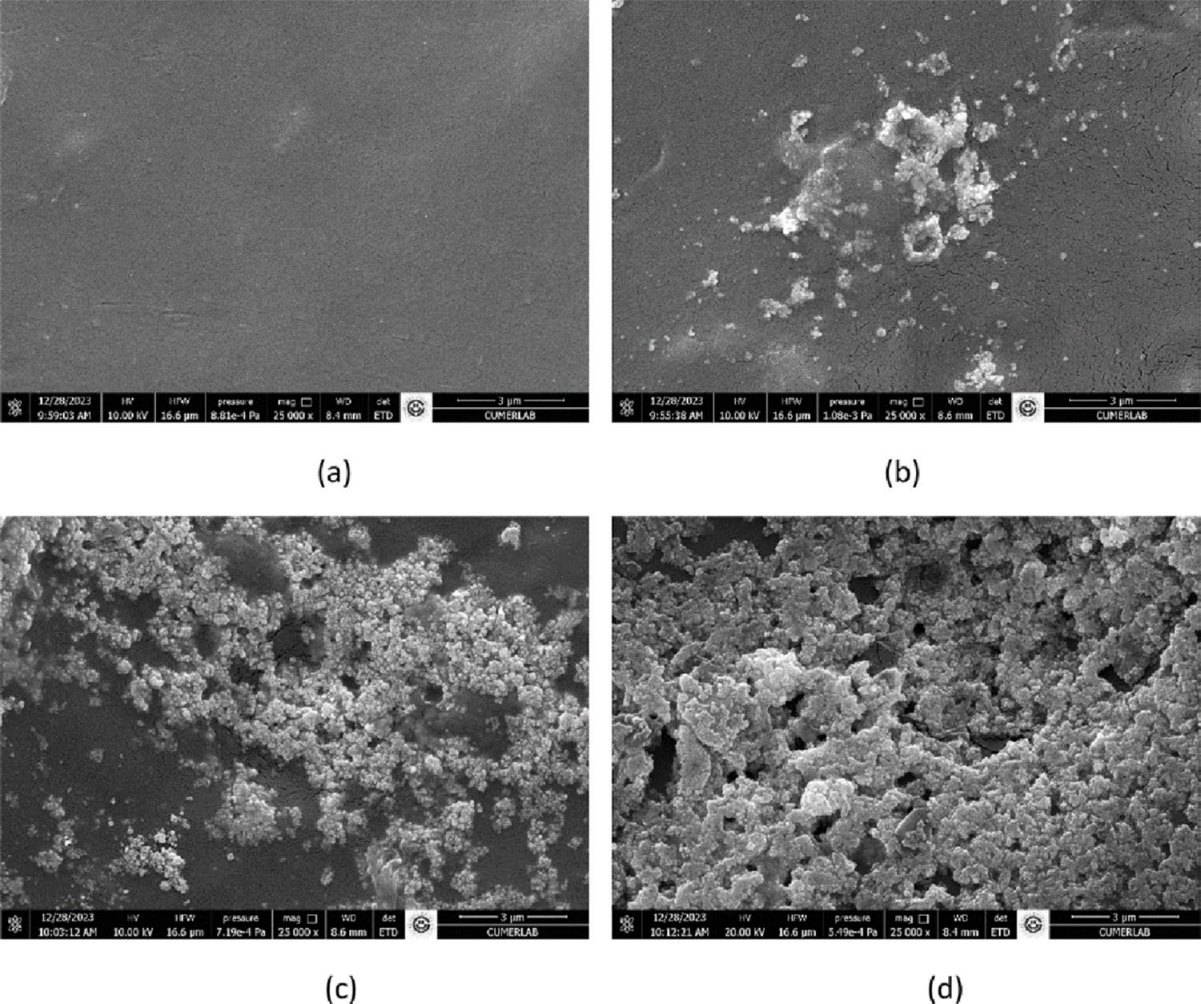
Scanning electronic microscopy (SEM) surface images of the samples; **a**) Standard sample, **b**) 1% wt. Bi_2_O_3_, **c**) 3% wt. Bi_2_O_3_, **d**) 5% wt Bi_2_O_3_. Magnification: 25,000

**Fig. 4:**
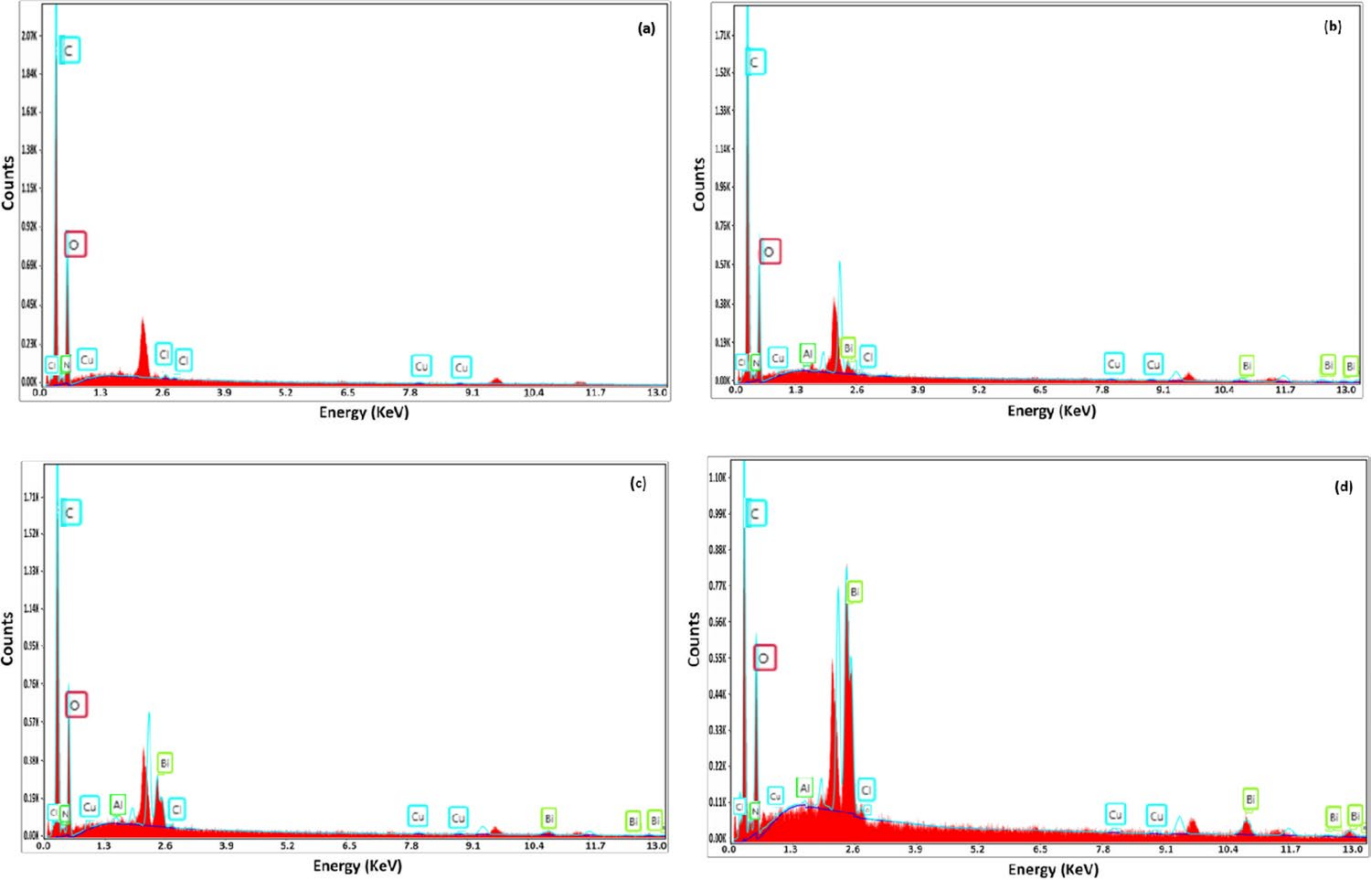
Energy dispersive X-ray spectroscopy (EDS) graphs: **a**) Standard sample, **b**) 1% wt. Bi_2_O_3_, **c**) 3% wt. Bi_2_O_3_, **d**) 5% wt. Bi_2_O_3_

The effect of Bi_2_O_3_ nanoparticle content on the surface morphology of the photopolymer composites was investigated using SEM and EDS. SEM images recorded at a magnification of 25,000× are presented in Fig. 3, while the corresponding EDS spectra are shown in Fig. 4.

SEM observations revealed that Bi_2_O_3_ nanoparticles were distributed relatively homogeneously within the photopolymer matrix at low reinforcement levels. In the samples containing 1 wt% Bi_2_O_3_, no pronounced agglomeration was observed, and the nanoparticles appeared to be uniformly embedded in the resin matrix. A similar behaviour has been reported by Alshahri et al. (2021), who observed homogeneous dispersion and preserved surface morphology in low density polyethylene (LDPE) based systems at low Bi_2_O_3_ contents. At intermediate reinforcement levels (approximately 3 wt%), small micro-cluster formations became visible on the surface. However, these clusters were limited in size and distribution, and the overall structure still retained a relatively homogeneous appearance. Comparable observations were reported by Alruwaili and El Sayed (2024), who noted the onset of localized agglomeration and a moderate increase in surface roughness at similar filler contents.

In contrast, samples with higher Bi_2_O_3_ content (5 wt%) exhibited pronounced agglomeration, as clearly evidenced by large and densely packed nanoparticle clusters in the SEM images (Fig. 3d). These regions appeared brighter and more compact due to the higher atomic number and density of bismuth. Marshall et al. (2025) reported a similar behaviour at elevated Bi_2_O_3_ loadings, where extensive agglomeration led to heterogeneous structures and deterioration of surface integrity. In the present study, increased surface roughness and morphological heterogeneity were evident at high reinforcement levels, indicating that excessive Bi_2_O_3_ content adversely affects surface uniformity.

EDS analyses confirmed the presence of carbon, oxygen, and bismuth in the reinforced samples, while no bismuth signal was detected in the reference sample without additive. At low reinforcement levels, elemental distribution was relatively uniform, whereas at higher Bi_2_O_3_ contents, bismuth was found to be concentrated in localized regions, consistent with the agglomeration zones observed in SEM images. Similar trends were reported by Ambika et al. (2017), who observed increased local bismuth enrichment with increasing filler content.

Quantitative EDS results showed that the detected Bi_2_O_3_ contents were significantly higher than the nominal reinforcement ratios, particularly at higher loadings, as summarized in Table 4. This discrepancy can be attributed to the inherent characteristics of the SLA process. In SLA systems, UV curing occurs from the bottom of the resin vat, and high-density Bi_2_O_3_ nanoparticles (~ 8.9 g·cm^− 3^) tend to sediment toward the lower regions of the resin under gravitational forces during fabrication. As a result, particle concentration increases near the surfaces that are cured first by UV exposure. When EDS analysis is performed on these particle-enriched regions, locally elevated bismuth contents are detected. For example, a Bi_2_O_3_ content of 26.78% was measured in the sample nominally containing 5 wt% additive, while a value of 6.96% was obtained for the 3 wt% sample.

**Table 4:** Bi_2_O_3_ percentages detected in energy dispersive X-ray spectroscopy (EDS)

Samples	Nominal Rate (%)	Detected by EDX (%)
Standard	0	0.00
1% wt.	1	1.65
3% wt.	3	6.96
5% wt.	5	26.78

These findings highlight the challenges associated with maintaining stable nanoparticle dispersion in SLA-based photopolymerization systems, particularly when heavy metal oxide fillers are used. Sedimentation and local particle enrichment contribute to surface heterogeneity and agglomeration at higher filler contents. Consequently, the Bi_2_O_3_ reinforcement level must be carefully optimized to balance homogeneous dispersion and functional performance. The present results, together with previous literature, indicate that low to moderate Bi_2_O_3_ contents provide a more uniform surface morphology, whereas excessive loading leads to agglomeration and deterioration of surface integrity.

### Electromagnetic shielding effectiveness

**Fig. 5:**
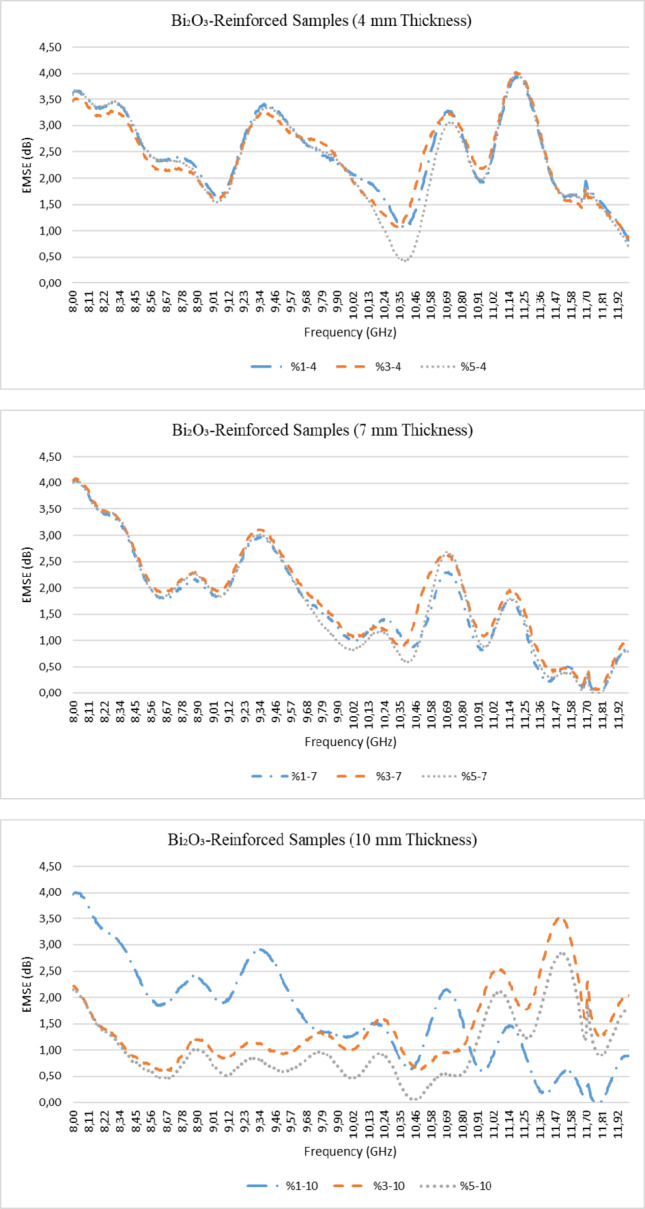
Electromagnetic shielding effectiveness (EMSE) results of Bi_2_O_3_-reinforced samples of various thickness

The electromagnetic shielding effectiveness (EMSE) of the Bi_2_O_3−_reinforced photopolymer nanocomposites were measured in the X-band frequency range (8–12 GHz), and the results are presented in Fig. 5. Overall, the measured EMSE values were low for all compositions and sample thicknesses investigated.

As shown in Fig. 5, polymer composites containing 1, 3, and 5 wt% Bi_2_O_3_ exhibited consistently weak electromagnetic shielding performance, regardless of sample thickness (4, 7, and 10 mm). For all tested conditions, the maximum EMSE values remained below 5 dB across the entire frequency range. Increasing either the Bi_2_O_3_ content or the sample thickness did not result in a meaningful improvement in shielding performance. These results clearly demonstrate that Bi_2_O_3_, when used as the sole filler in an electrically insulating polymer matrix, does not contribute to the attenuation of electromagnetic radiation.

The observed behaviour is consistent with previous findings reported in the literature. Bi_2_O_3_ is known to exhibit low electrical conductivity and primarily acts as a dielectric material with limited electromagnetic wave absorption capability (Liu and Yang 2021; Maestre and Santos 2023). The absence of electrically conductive pathways within the composite prevents the formation of effective reflection- or absorption-dominated shielding mechanisms. Maestre and Santos (2023) reported that even in geopolymer-based systems containing 5 wt% Bi_2_O_3_, the improvement in EMSE was modest, increasing only from 21.2 dB to 23.6 dB, and this enhancement was attributed to improved dispersion within a rigid matrix. In contrast, the present photopolymer-based system lacks both intrinsic conductivity and matrix rigidity, which further limits shielding performance. Similarly, Liu and Yang (2021) demonstrated that effective EMSE was achieved only at very high total filler contents (approximately 45 wt%) through the combined use of graphite, bismuth, and bismuth oxide, whereas Bi_2_O_3_ alone resulted in weak shielding behaviour.

Overall, the present results confirm that Bi_2_O_3_ alone is not effective filler for electromagnetic interference shielding in polymer-based systems. Without the incorporation of conductive additives or the formation of a percolated conductive structure, Bi_2_O_3_ filled photopolymer composites remain functionally limited for electromagnetic shielding applications, even at increased filler loadings and sample thicknesses.

### Surface resistivity

Surface resistivity measurements showed that all SLA-printed nanocomposite samples containing 1, 3, and 5 wt% Bi_2_O_3_ exhibited values exceeding the upper measurement limit of the measurement device (> 10¹² Ω/sq). Therefore, exact surface resistivity values could not be determined. Based on commonly accepted surface resistivity classification ranges reported in the literature, all samples were classified as electrically insulating (Groop et al. 2003). This insulating behaviour was consistent across all investigated Bi_2_O_3_ loadings.

This electrical behaviour directly explains the observed limited electromagnetic shielding effectiveness (EMSE) described in the previous section. Electromagnetic shielding relies primarily on reflection and absorption mechanisms, both of which are strongly enhanced by sufficient electrical conductivity. In insulating systems, reflection-based shielding is largely suppressed due to the lack of free charge carriers, and absorption losses remain minimal in the absence of conductive or magnetic filler networks. As a result, the formation of an effective shielding structure is not possible without a percolated conductive pathway.

Nan et al. emphasized that the absence of a conductive filler phase prevents the formation of a percolation network, resulting in negligible shielding performance even at increased filler loadings (Nan et al. 2024). Likewise, Shukla reported that significant improvements in shielding effectiveness were achieved only after incorporating conductive nanoparticles, such as iron-based fillers, into otherwise insulating polymer matrices (Shukla 2019).

In the present study, increasing the Bi_2_O_3_ content up to 5 wt% did not lead to meaningful improvements in surface conductivity or shielding performance, confirming that Bi_2_O_3_ alone cannot provide the electrical pathways required for effective electromagnetic shielding. It should be emphasized that electromagnetic shielding and ionizing radiation shielding rely on different mechanisms. In the present system, Bi_2_O_3_ improved ionizing radiation attenuation because of the high atomic number of bismuth (see below), whereas electromagnetic shielding remained weak due to the electrically insulating nature of both the filler and the photopolymer matrix. These findings highlight the necessity of incorporating conductive fillers or using intrinsically conductive polymer systems when electromagnetic interference shielding is desired in SLA-fabricated nanocomposites.

### Antibacterial activity

All Bi_2_O_3_-reinforced SLA nanocomposite surfaces containing 1 wt%, 3 wt%, and 5 wt% Bi_2_O_3_ exhibited strong antibacterial activity against *E. coli* and *S. aureus*, with bacterial reduction rates exceeding 99% after 24 h of contact according to ISO 22,196 (ISO, 2011). No detectable bacterial colonies were observed after 24 h for any of the Bi_2_O_3_-containing samples. Therefore, all samples were evaluated as exhibiting antibacterial activity greater than 99%. According to the standard classification, this level of reduction corresponds to strong antibacterial performance. This antibacterial behaviour was consistent across all reinforcement levels, indicating that even the lowest Bi_2_O_3_ content provided sufficient antibacterial functionality. In contrast to the Bi₂O₃-reinforced samples, the reference sample (0 wt% Bi₂O₃) showed no significant antibacterial effect, and visible bacterial colony formation remained after 24 h of incubation. This result confirms that the antibacterial activity observed in the composites was directly associated with the incorporation of Bi₂O₃ nanoparticles.

**Fig. 6:**
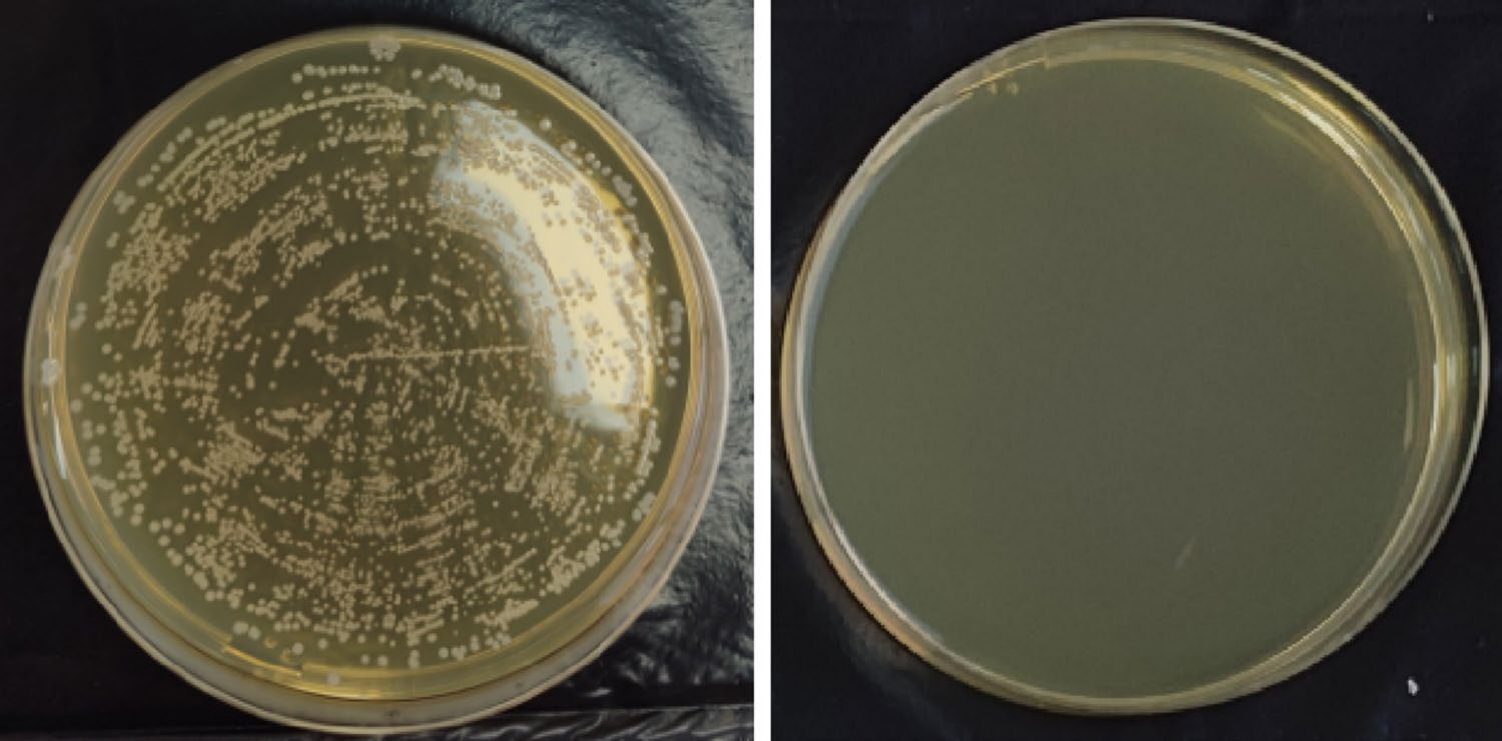
Representative Petri dish images showing bacterial colony formation immediately after inoculation (0 h, left) and after 24 h of contact (right) with the Bi_2_O_3_-reinforced SLA surface

Representative Petri dish images are presented in Fig. 6, showing bacterial colonies immediately after inoculation (0 h) and after 24 h of contact with the Bi_2_O_3_ containing SLA surfaces. Dense colony formation was observed at 0 h, whereas no visible colonies were detected after 24 h, confirming the near-complete inactivation of viable bacterial cells.

The pronounced antibacterial performance of Bi_2_O_3_ is closely related to its distinctive mechanism of action. Unlike silver- or copper-based antimicrobial systems, which primarily rely on reactive oxygen species generation or membrane disruption, bismuth compounds mainly interfere with bacterial metabolic pathways. Bi_2_O_3_ exhibits antibacterial activity mainly through interference with bacterial metabolic processes rather than through membrane damage or reactive oxygen species generation. Bismuth compounds are known to target key enzymatic pathways involved in cellular respiration, including the tricarboxylic acid cycle and oxidative phosphorylation, leading to impaired energy production and eventual cell death. Bismuth-based drugs disrupt multiple essential pathways in bacterial cells by damaging oxidative defense systems and eliminating pH-buffering capability through binding to and functional perturbation of several key enzymes (Rosário et al. 2023; Li et al. 2025).

Recent studies support the present findings. Pant et al. (2022) reported that α-Bi_2_O_3_ nanoparticles exhibited minimum inhibitory concentration values as low as 0.75 µg/mL against *P. aeruginosa* and 2.5 µg/mL against *S. aureus*. Similarly, Geoffrion et al. (2021) demonstrated that Bi_2_O_3_ nanostructures were capable of completely suppressing bacterial growth at concentrations of approximately 50 ppm. These results are consistent with the present study, where even 1 wt% Bi_2_O_3_ reinforcement was sufficient to fully suppress bacterial colony formation after 24 h of contact.

Overall, the ISO 22,196 results clearly demonstrate that Bi_2_O_3_ is a highly effective antibacterial additive for SLA-printed nanocomposites. The combination of strong antibacterial performance, stability, and low toxicity highlights the potential of Bi_2_O_3_-reinforced photopolymer composites for applications requiring hygienic, medical, or antimicrobial surface properties.

### Ionizing radiation shielding results

The calculated mass attenuation coefficient (µ/ρ) values of the Bi_2_O_3_-reinforced SLA composites at different photon energies are summarized in Table 5.

**Table 5:** µ/ρ (cm^2^/g) Values determined using the WinXCOM and EpiXS codes

Energy (MeV)	0% Bi_2_O_3_		1% Bi_2_O_3_		3% Bi_2_O_3_		5% Bi_2_O_3_	
	WinXCOM	EpiXS	WinXCOM	EpiXS	WinXCOM	EpiXS	WinXCOM	EpiXS
0.015	1.1297	1.1308	2.1600	2.1599	4.2231	4.2206	6.2827	6.2777
0.020	0.5829	0.5839	1.3805	1.3790	2.9778	2.9712	4.5722	4.5606
0.030	0.3059	0.3062	0.5858	0.5864	1.1463	1.1475	1.7058	1.7076
0.040	0.2356	0.2358	0.3676	0.3678	0.6319	0.6319	0.8957	0.8957
0.050	0.2073	0.2074	0.2806	0.2804	0.4273	0.4267	0.5737	0.5726
0.060	0.1920	0.1920	0.2372	0.2368	0.3277	0.3267	0.4180	0.4163
0.080	0.1745	0.1744	0.1955	0.1949	0.2376	0.2360	0.2797	0.2770
0.100	0.1634	0.1633	0.2134	0.2131	0.3134	0.3128	0.4133	0.4123
0.150	0.1449	0.1449	0.1623	0.1622	0.1971	0.1970	0.2318	0.2316
0.200	0.1322	0.1322	0.1403	0.1402	0.1564	0.1564	0.1726	0.1726
0.300	0.1146	0.1146	0.1173	0.1173	0.1227	0.1227	0.1281	0.1281
0.400	0.1026	0.1025	0.1038	0.1037	0.1062	0.1062	0.1087	0.1086
0.500	0.0936	0.0936	0.0943	0.0942	0.0955	0.0955	0.0968	0.0967
0.600	0.0866	0.0865	0.0869	0.0869	0.0877	0.0876	0.0884	0.0883
0.800	0.0760	0.0760	0.0761	0.0761	0.0764	0.0763	0.0766	0.0766
1.000	0.0684	0.0683	0.0684	0.0683	0.0684	0.0684	0.0685	0.0684
1.500	0.0556	0.0556	0.0556	0.0555	0.0555	0.0555	0.0555	0.0554
2.000	0.0477	0.0476	0.0477	0.0476	0.0477	0.0476	0.0477	0.0476
3.000	0.0383	0.0382	0.0383	0.0382	0.0384	0.0383	0.0384	0.0384
4.000	0.0327	0.0326	0.0328	0.0327	0.0330	0.0329	0.0331	0.0331
5.000	0.0291	0.0290	0.0292	0.0291	0.0294	0.0294	0.0297	0.0296
6.000	0.0265	0.0264	0.0266	0.0266	0.0270	0.0269	0.0273	0.0272
8.000	0.0231	0.0230	0.0233	0.0233	0.0237	0.0237	0.0242	0.0241
10.000	0.0210	0.0209	0.0213	0.0212	0.0218	0.0217	0.0223	0.0222
15.000	0.0182	0.0181	0.0185	0.0185	0.0192	0.0192	0.0199	0.0199

**Fig. 7:**
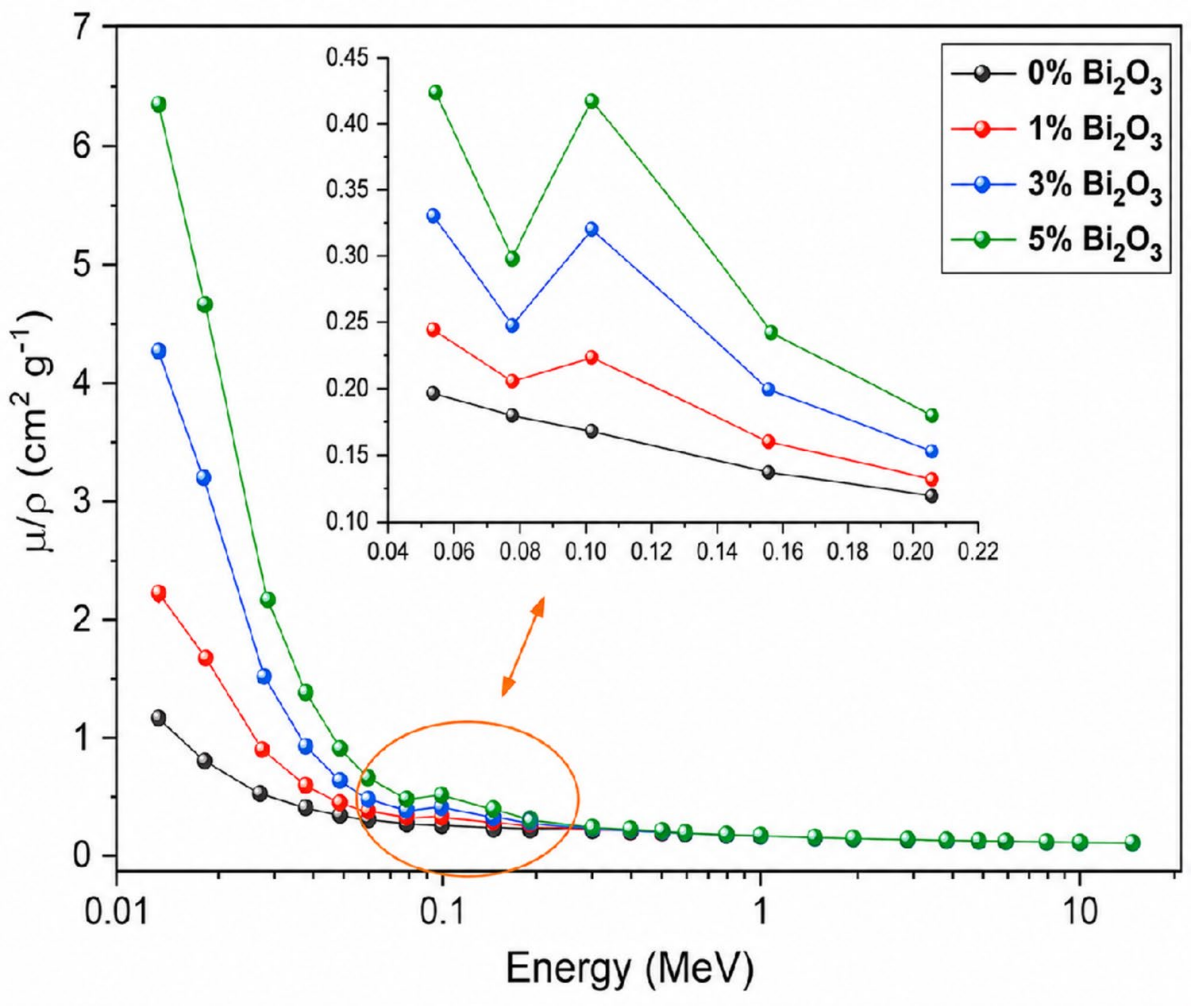
Mass attenuation coefficient (µ/ρ) versus photon energy

As presented in Table 5, the mass attenuation coefficient (µ/ρ) values increase systematically with increasing Bi_2_O_3_ content across the entire photon energy range. The highest µ/ρ values were obtained for the samples containing 5 wt% Bi_2_O_3_, while the lowest values correspond to the unfilled photopolymer. This enhancement is more pronounced at low photon energies, where photon interaction is dominated by the photoelectric effect. In this region, the probability of photoelectric absorption strongly depends on the atomic number (Z) of the absorber. Since bismuth is a high-Z element, the incorporation of Bi_2_O_3_ significantly enhances photon interaction and absorption at low energies. Therefore, the beneficial effect of Bi_2_O_3_ reinforcement becomes most evident in the low-energy region. As the photon energy increases, µ/ρ values decrease for all samples, and the difference between different Bi_2_O_3_ loadings becomes less distinct due to the transition from photoelectric absorption to Compton scattering. The variations of µ/ρ with photon energy for all samples are given in Fig. 7.

In all cases µ/ρ peaks at the lowest energies (~ 0.01–0.1 MeV) and falls rapidly with increasing energy. The energy dependence of the shielding parameters can be interpreted in three distinct regions. In the low-energy region, attenuation is mainly governed by the photoelectric effect, and therefore Bi_2_O_3_ containing samples exhibit a clear advantage due to the high atomic number of bismuth. In the intermediate-energy range, Compton scattering becomes dominant, and the differences between compositions decrease because this interaction depends less strongly on atomic number. At higher photon energies, attenuation continues to decrease, and the contribution of pair production may begin above 1.022 MeV, resulting in flatter trends and smaller relative differences among the samples. This behaviour reflects the dominance of photoelectric absorption at low energies (µ/ρ roughly ∝ E^− 3.5^) and a transition to Compton scattering at intermediate energies (µ/ρ ∼ E^− 1^) (Özdoğan et al. 2024). Consistent with prior studies, adding Bi_2_O_3_ (a high–atomic-number, high-density filler) substantially raises µ/ρ at all energies. For example, at ~ 0.06 MeV the 5 wt% Bi_2_O_3_ sample has the highest µ/ρ, followed by 3 wt%, 1 wt%, and the reference samples. The differences shrink at higher energies, but even at 0.1 MeV the Bi_2_O_3_ composites remain more attenuating than the reference sample. This behaviour around 0.1 MeV can be attributed to the transition from photoelectric absorption to Compton scattering, where the dominant interaction mechanism changes and affects the attenuation trend. In addition, the K-shell absorption edge energy of the Bi (E_KBi_ = 90.536 keV) element is present in this region. Since photoelectric absorption is more dominant in the absorption edge energies of the shells, such fluctuations occur. These trends agree with results reported by Hamada et al. (2025) and Shabib et al. (2025), who observed increased γ-ray attenuation with increasing Bi_2_O_3_ content in polymer matrices (Hamada et al. 2025; Shabib et al. 2025). Similar observations have also been reported in previous studies on Bi_2_O_3_-based polymer composites, where increasing Bi_2_O_3_ content results in higher mass attenuation coefficients, reduced half-value layer (HVL) values, and improved overall shielding performance. These findings further confirm the consistency of the present results with the existing literature (Ambika et al. 2017; Alruwaili and El Sayed 2024).

**Fig. 8:**
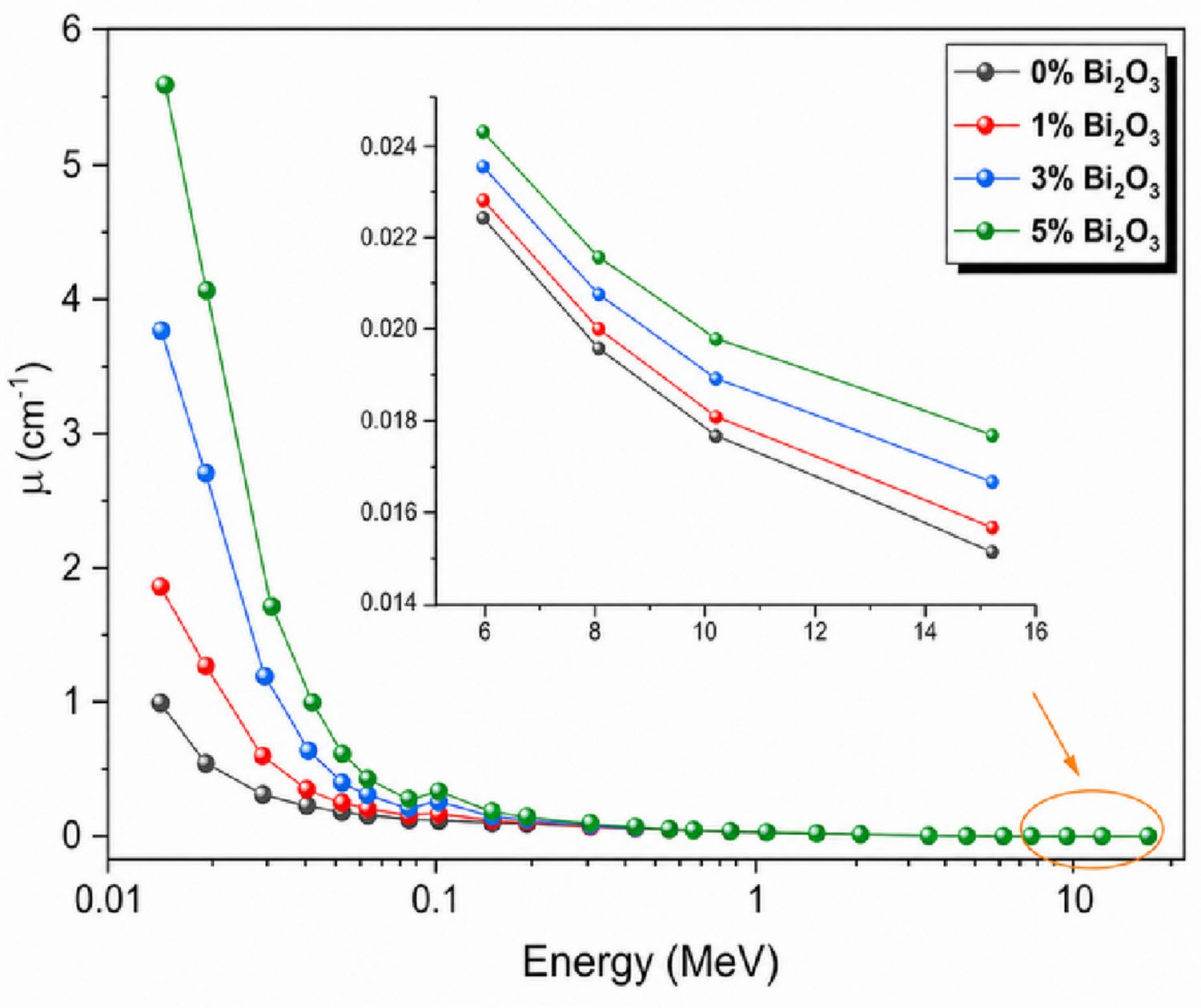
Linear attenuation coefficient (µ) versus photon energy

Similarly, the linear attenuation coefficient (µ) curves in Fig. 8 mirror the µ/ρ trends but include the effect of composite density. The Bi_2_O_3_-reinforced samples have higher density, so their µ values exceed those of the pure polymer by an even larger margin. Thus the 5% Bi_2_O_3_ composite has the largest µ at every energy, reflecting both higher µ/ρ and higher mass density. Overall, increasing Bi_2_O_3_ loading enhances photon attenuation (higher µ/ρ and µ), as noted in previous studies (Hamada et al. 2025; Shabib et al. 2025).

**Fig. 9:**
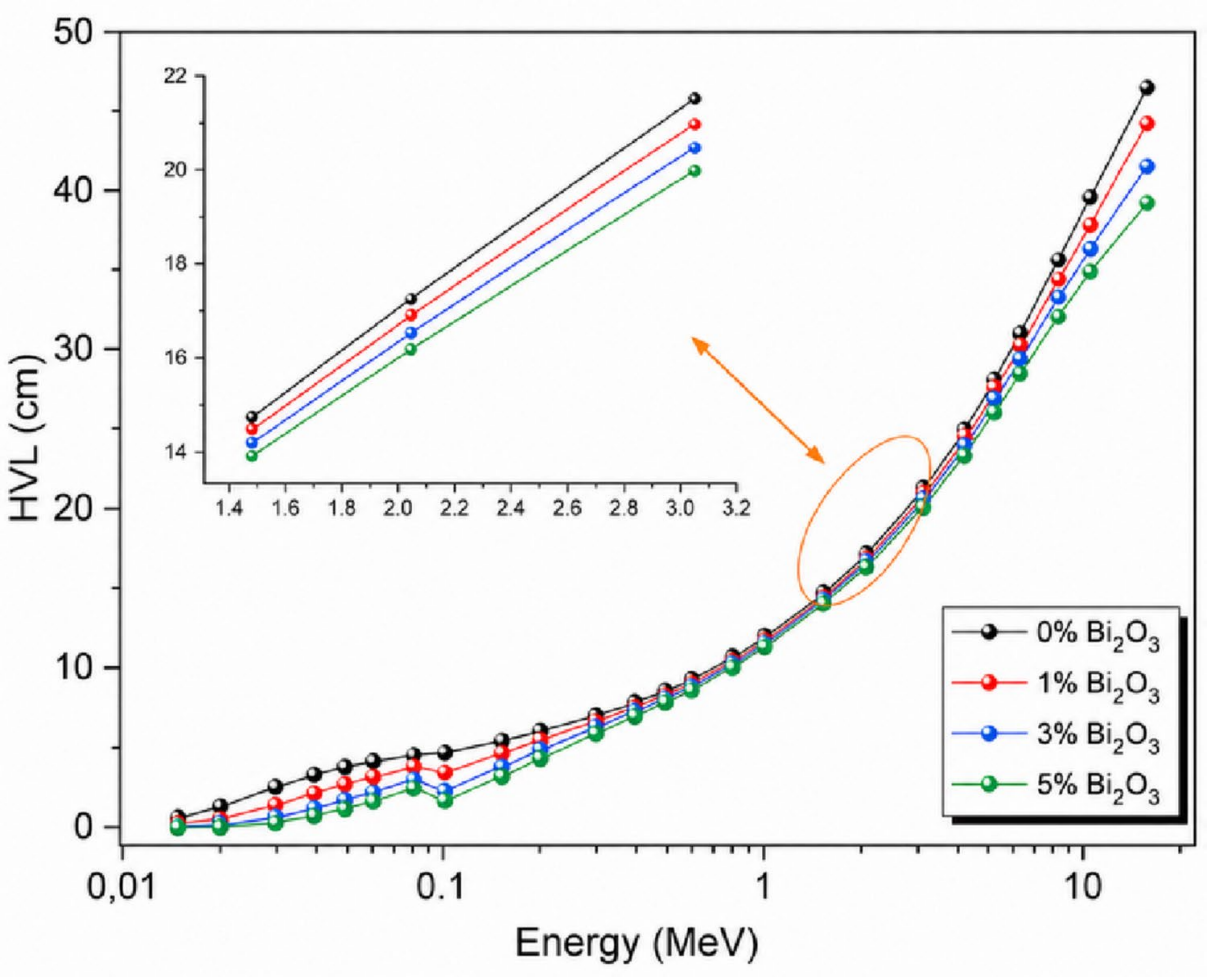
Half-value layer (HVL) versus photon energy

HVL is inversely related to µ: materials with stronger attenuation (higher µ) require less thickness to halve the intensity. The lower HVL values obtained for the Bi_2_O_3_-containing samples indicate that the same attenuation level can be achieved with a smaller material thickness. Likewise, the higher RPE values show that increasing Bi_2_O_3_ content improves shielding efficiency at a fixed sample thickness. The HVL versus photon energy graph for each composite is shown in Fig. 9. As expected, HVL is smallest at low photon energies and grows with energy. For all materials, HVL rises sharply from ~ 0.01 MeV to ~ 0.1 MeV (photoelectric-dominated range) and continues to increase more gradually into the MeV range. Crucially, at every energy the HVL of Bi2O3-filled composites are lower than that of the pure resin. The 5% Bi_2_O_3_ specimen consistently has the lowest HVL, followed in order by 3%, 1%, and 0%. In other words, higher Bi_2_O_3_ content yields thinner required shields (lower HVL), confirming that the filler markedly improves shielding. For instance, at low energies the HVL of the 5% composite is reduced by ~ 40–60% relative to pure SLA, in agreement with Hamada et al. (2025) who also reported HVL reductions with Bi_2_O_3_ addition (Hamada et al. 2025). In practical terms, this means that adding a few percent of Bi_2_O_3_ can significantly reduce the material thickness needed for a given attenuation.

Across all compositions, HVL climbs with photon energy. This reflects the same physics: as energy increases into the Compton scattering, µ decreases (fewer photon interactions), so more material is needed to attenuate 50% of the beam. At energies above 1.022 MeV, pair production begins to contribute, causing µ to flatten and HVL growth to slow (Abbas et al. 2023). In the present data, beyond about 1–2 MeV the HVL curves begin to level out, indicating the pair production threshold.

**Fig. 10:**
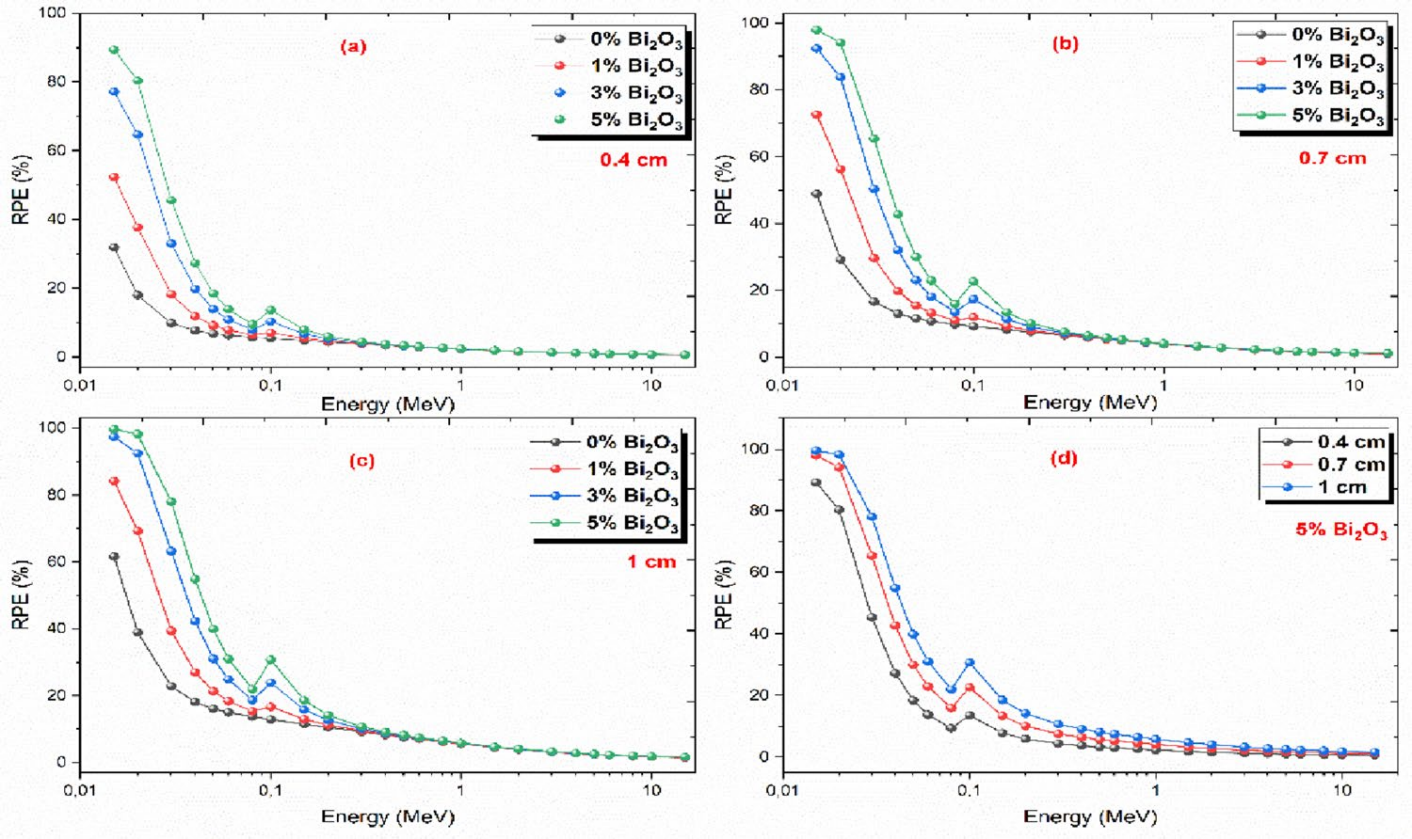
Radiation protection efficiency (RPE) of the investigated samples; **a**) 0.4 cm thickness; **b**) 0.7 cm thickness; **c**) 1 cm thickness; **d**)

RPE represents the fraction of incident photons removed by the material. Figure 10 shows the calculated RPE values of the investigated samples. As expected, RPE is very high (often > 90–99%) at the lowest photon energies for all samples, and then drops as energy increases. For example, at 0.01–0.05 MeV, nearly all photons are stopped even in the pure matrix; in this photoelectric event the Bi_2_O_3_-filled composites essentially reach maximal efficiency. However, above a few hundred keV (as Compton scattering takes over), RPE falls off significantly. The data show that the 5% Bi_2_O_3_ composite maintains the highest RPE at each energy. At intermediate energies (0.1–1 MeV), the gap in RPE between different Bi_2_O_3_ loadings narrows, but remains appreciable: e.g., at about 0.06 MeV the 5% composite might shield roughly twice the fraction of photons compared to the 0% sample. Similar behaviour was reported by Özdoğan et al. (2024), who observed that polymer composites exhibited maximum RPE values in the low-energy gamma region, with the strongest shielding occurring below approximately 0.06 MeV. Above about 1 MeV, RPE values for all samples converge at modest levels (tens of percent), since attenuation is inherently weaker. This behaviour of a high RPE at low energy falling steeply around ~ 0.1–0.3 MeV exactly parallels earlier findings: in photoelectric-dominated regions RPE is large and very different between compositions, while in the Compton region RPE declines and differences shrink (Özdoğan et al. 2024). These results indicate that the shielding performance of the developed composites is most effective in the low photon energy region, particularly below approximately 0.1 MeV.

Across all shielding metrics, the performance trend with composition is clear: 5% Bi_2_O_3_ > 3% Bi_2_O_3_ > 1% Bi_2_O_3_ > 0% Bi_2_O_3_. Even the 1% composite outperforms the neat resin, demonstrating that even small amounts of high‑Z filler are effective. The literature agrees that attenuation coefficients (and efficiencies) increase monotonically with Bi_2_O_3_ loading (Hamada et al. 2025; Shabib et al. 2025).

While Bi_2_O_3_/SLA nanocomposites offer improved γ‑ray shielding, there are practical limits. At high photon energies (multi‑MeV range), attenuation remains relatively poor, so very thick sections would be needed for significant protection. In other words, the material’s performance is best in the photoelectric regime; in the pair production regime the benefit of modest Bi_2_O_3_ loading is limited (Abbas et al. 2023; Özdoğan et al. 2024). Additionally, adding large amounts of filler can impair other properties. Hamada et al. observed that at 20 wt% Bi_2_O_3_ the composite’s compressive strength dropped by about 50% (Hamada et al. 2025). Even though the highest loading in the present study was lower, this underscores that mechanical strength, ductility or processability can suffer at high filler content. Thus, design of a practical shield must balance shielding gain against factors like weight, thickness, flexibility and cost.

In summary, the present results supported by recent studies – show that small Bi_2_O_3_ additions significantly enhance SLA polymer shielding at low to intermediate γ energies (Özdoğan et al. 2024; Hamada et al. 2025).

## Conclusions

In this study, Bi_2_O_3_-reinforced photopolymer nanocomposite surfaces were produced using stereolithography (SLA) and evaluated in terms of surface morphology, electrical behaviour, electromagnetic shielding, antibacterial activity, and ionizing radiation shielding performance. The results show that SLA is a suitable method for fabricating functional photopolymer surfaces when appropriate inorganic fillers are used.

Morphological analysis indicated that Bi_2_O_3_ nanoparticles were well distributed within the photopolymer matrix at low filler contents. At higher Bi_2_O_3_ loadings, particle agglomeration and surface heterogeneity became more pronounced, mainly due to sedimentation during the SLA process. This highlights the need for careful control of filler content and dispersion when heavy metal oxide particles are added to low-viscosity photopolymer resins.

All Bi_2_O_3_-containing samples showed strong antibacterial activity, with bacterial reduction rates higher than 99% against E. coli and S. aureus after 24 h of contact. Notably, this high antibacterial performance was achieved even at the lowest filler content of 1 wt%, confirming that Bi_2_O_3_ is an effective and stable antibacterial additive for SLA-printed surfaces.

Surface resistivity measurements demonstrated that all nanocomposites remained electrically insulating, with resistivity values above 10^12^ Ω/sq. As a result, electromagnetic shielding effectiveness remained below 5 dB in the X-band frequency range. These findings clearly show that Bi_2_O_3_ alone cannot provide effective electromagnetic shielding in insulating polymer matrices and that addition of conductive filler is required for such applications.

In contrast, theoretical ionizing radiation shielding calculations revealed that Bi_2_O_3_ significantly improves photon attenuation, especially at low photon energies. The shielding improvement was found to be most pronounced in the low photon energy region, particularly below approximately 0.1 MeV. It should also be noted that electromagnetic shielding and ionizing radiation shielding are governed by different mechanisms; while Bi_2_O_3_ enhances ionizing radiation attenuation due to its high atomic number, it does not contribute to electromagnetic shielding because of its electrically insulating nature. Higher Bi_2_O_3_ contents lead to increased attenuation coefficients, lower half-value layer values, and higher radiation protection efficiency. This behaviour is mainly attributed to the high atomic number of bismuth, which enhances photon interaction at low energies.

Overall, Bi_2_O_3_-reinforced SLA nanocomposites combine strong antibacterial performance, electrical insulation, and improved ionizing radiation shielding, while showing limited electromagnetic shielding capability. These properties make the materials promising for hygienic and radiation-related surface applications, particularly in medical and laboratory environments. Future work may focus on improving particle dispersion and exploring hybrid filler systems to further enhance the functional performance of SLA-printed nanocomposites.

## Data Availability

The data that support the findings of this study are available from the corresponding author upon reasonable request.
